# White Cord Syndrome: A Treatment Dilemma

**DOI:** 10.7759/cureus.38177

**Published:** 2023-04-26

**Authors:** Leong Yen Hsin, Vijay Vengkat Samynathan C, Huang Yilun

**Affiliations:** 1 Department of Orthopaedic Surgery, Sengkang General Hospital, Singapore, SGP; 2 Department of Orthopaedic Surgery, Lee Kong Chian School of Medicine, Singapore, SGP

**Keywords:** neurological deterioration, rare complication, cervical decompressive surgery, reperfusion injury, white cord syndrome

## Abstract

Spinal cord reperfusion injury following decompressive surgery is extremely rare. This complication is known as white cord syndrome (WCS).

A 61-year-old male presented with chronic neck stiffness associated with left C6/C7 radiculopathy and numbness. Magnetic resonance imaging (MRI) of the cervical spine reported a severely narrowed left C6/C7 neural exit canal. C6/C7 anterior cervical decompression and fusion (ACDF) was performed. There was no significant intraoperative injury. On postoperative day 6, the patient developed bilateral C8 numbness, which started post-operation. He was treated for surgical site inflammation and was prescribed prednisolone and amitriptyline. However, his condition progressively worsened. At postoperative six weeks, there was right hemisensory loss, right triceps atrophy, and positive right Lhermitte’s and Hoffman’s tests. This subsequently progressed to right C7 weakness and bilateral lower limb radiculopathy at postoperative eight weeks. Postoperative MRI of the cervical spine revealed a new focal gliosis/edema within the spinal cord at C6/C7. The patient was treated conservatively with pregabalin and was referred for rehabilitation.

Early diagnosis and treatment initiation are crucial in the management of WCS. Surgeons should be aware of this potential complication and counsel patients on the risk prior to surgery. Magnetic resonance imaging (MRI) remains the gold standard in the diagnosis of WCS. The current mainstay of treatment is high-dose steroids, intraoperative neurophysiological monitoring, and early recognition of postoperative WCS.

## Introduction

White cord syndrome (WCS) is an extremely rare complication. It is thought to be a result of reperfusion injury following spinal decompressive surgery, causing neurological deterioration in the absence of perioperative injury [1]. The presence of intramedullary hyperintense area in postoperative T2-weighted magnetic resonance imaging (MRI), without other pathological signs, is the hallmark of WCS [2].

## Case presentation

A 61-year-old male presented with chronic neck stiffness associated with left C6/C7 radiculopathy and numbness. The symptoms were aggravated in a sitting position and relieved on lying down. He had no limb weakness, fever, night pain, weight loss, or upper back pain. On examination, there was no spinal tenderness. However, the cervical extension was limited, and Spurling’s test was positive on the left side. Power, sensation, and reflexes were normal. Hoffman’s test was negative, and tandem gait was normal.

He had a previous decompressive surgery done two years prior for left C6/C7 neural foramen stenosis.

**Figure 1:**
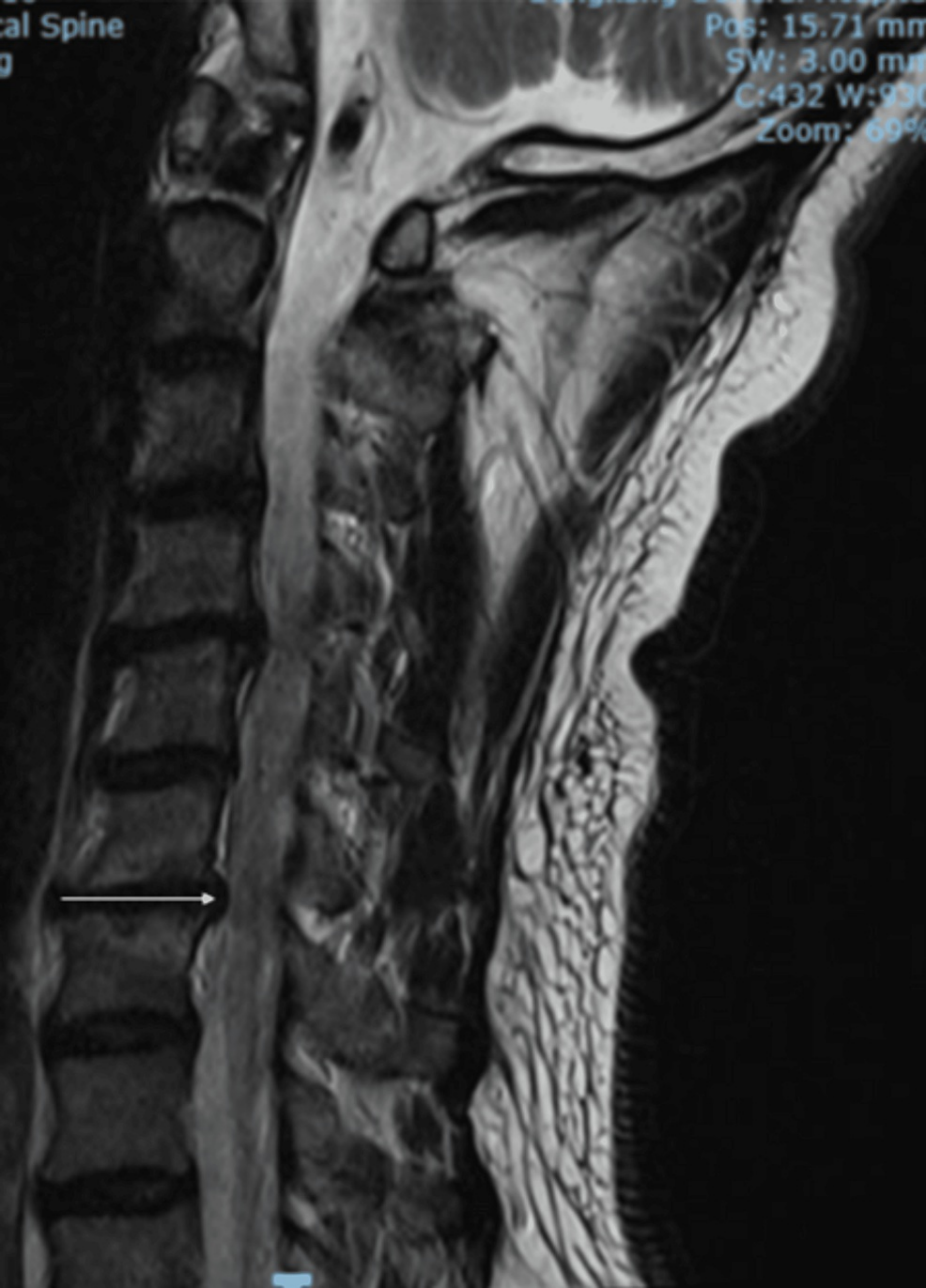
Preoperative T2-weighted MRI of the cervical spine (sagittal view) No spinal cord compression at C6/C7 (arrow) MRI: magnetic resonance imaging

**Figure 2:**
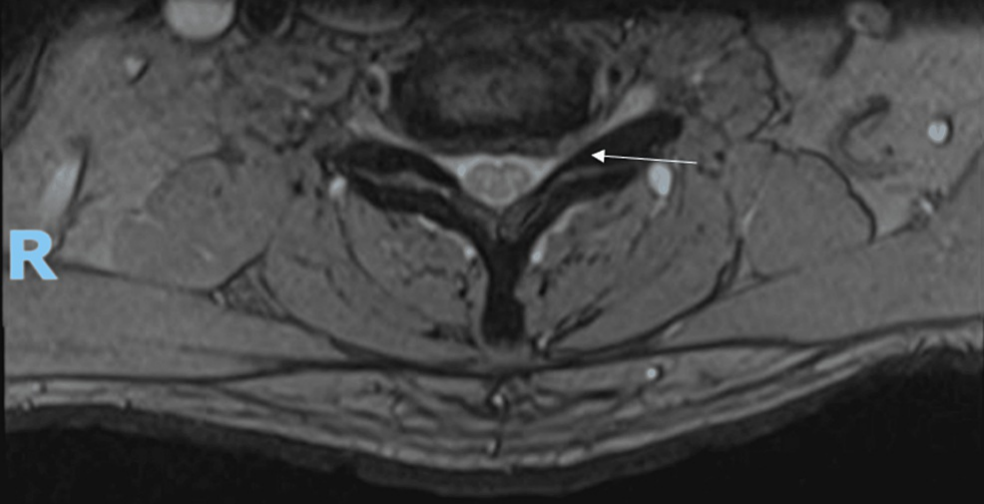
Preoperative T2-weighted MRI of the cervical spine (axial view) Severely narrowed left C6/C7 neural exit canal (arrow) MRI: magnetic resonance imaging

Magnetic resonance imaging (MRI) of the cervical spine reported a severely narrowed left C6/C7 neural exit canal and moderate spinal canal stenosis at C3/C4 and C4/C5. There was no spinal cord compression (Figures 1, 2). MRI of the thoracic spine was unremarkable. A nerve conduction study showed bilateral mild carpal tunnel syndrome worse over the left side.

Physiotherapy and acupuncture did not improve the symptoms. He subsequently underwent an uneventful C6/C7 anterior cervical decompression and fusion (ACDF). Intraoperatively, there was severe stenosis of the bilateral C6/C7 neural foramen. Intraoperative neuromonitoring did not show any alert. Postoperative radiographs were satisfactory.

He was readmitted on postoperative day 6 for new-onset bilateral C8 numbness, which started post-operation. There was no bowel bladder incontinence, loss of dexterity, or swallowing difficulty. Gait was steady, and bilateral upper limb power was intact. He was treated for surgical site inflammation and was discharged with prednisolone and amitriptyline.

Upon outpatient review at postoperative six weeks, the patient complained of right triceps wasting with a right hemisensory loss. Lhermitte’s and Hoffman’s tests were positive on the right side. The sensation was reduced over the right half of the body. Reflexes were equivocal. Gait was steady, and bilateral upper limb power was intact. There was no facial palsy, and the sensation over the face was intact. There were no syncopal attacks, headaches, neck aches, or backaches.

At postoperative eight weeks, he presented with bilateral lower limb radiculopathy. The radicular pain was aggravated by neck flexion and was associated with numbness over the right upper limb and tips of the left ring and little finger. He was still able to hold small objects. There was no bowel bladder dysfunction and no trauma prior to the onset of symptoms. On examination, there was right triceps muscle atrophy. Power was reduced over the right C7 region (Medical Research Council (MRC) grade 4/5). The sensation was intact. Hoffman’s and Lhermitte’s tests were positive on the right side. Reflexes were equivocal, and gait was steady. Bilateral lower limb power and sensation were intact. The anal tone was normal, and there was no saddle anesthesia.

**Figure 3:**
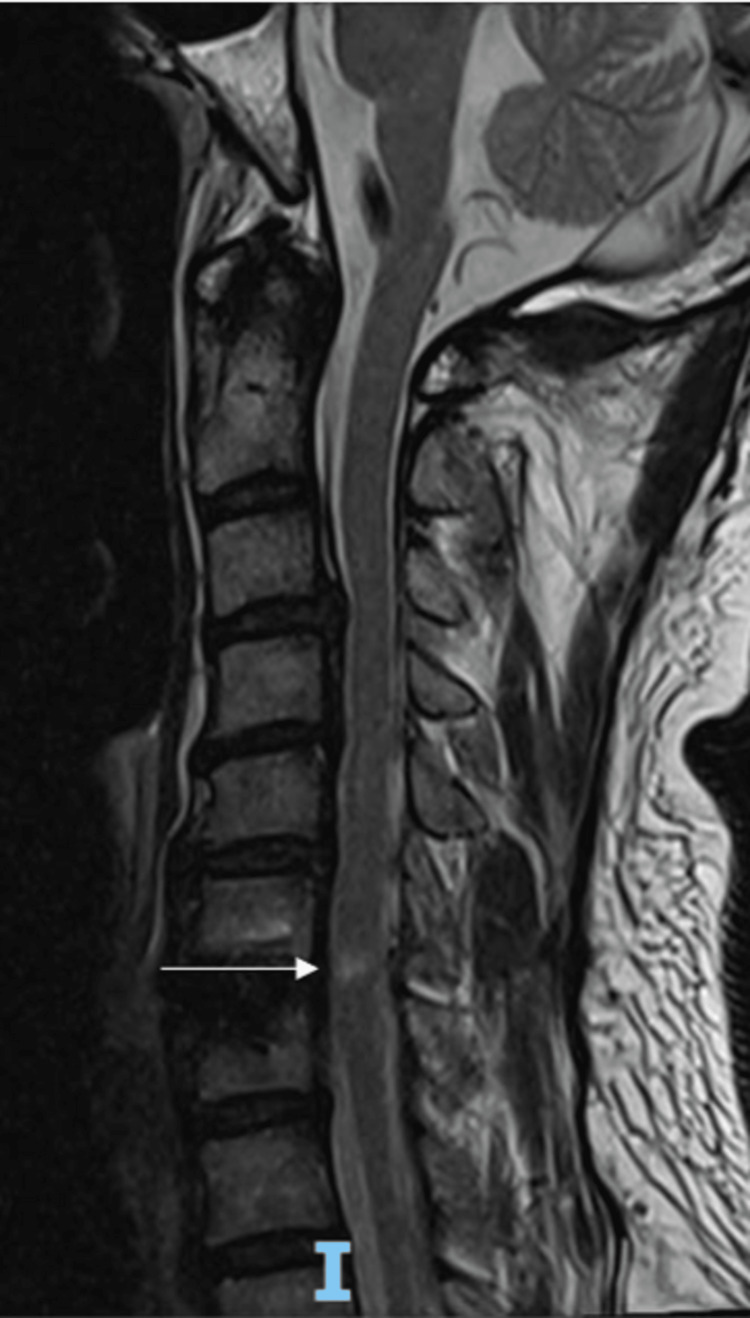
Postoperative T2-weighted MRI of the cervical spine (sagittal view) New focal gliosis/edema within the spinal cord at C6/C7 (arrow) MRI: magnetic resonance imaging

**Figure 4:**
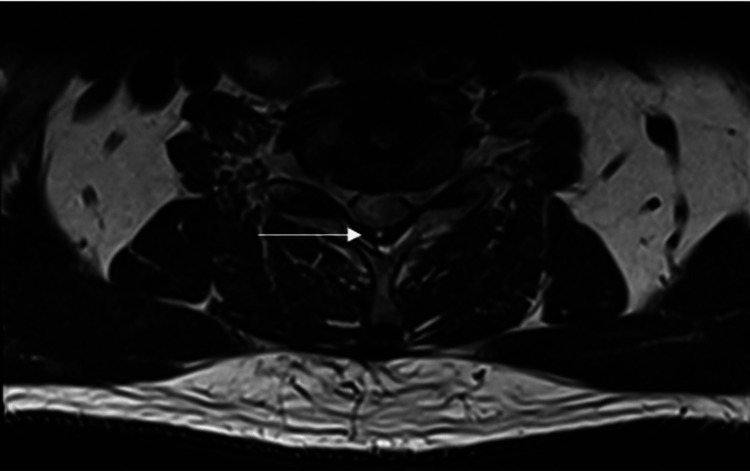
Postoperative T2-weighted MRI of the cervical spine (axial view) New focal gliosis/edema within the spinal cord at C6/C7 (arrow) MRI: magnetic resonance imaging

MRI of the cervical spine revealed a new focal gliosis/edema within the spinal cord at C6/C7. There was no cord compression, a foreign body within the theca, or abnormal enhancement (Figures 3, 4). MRI of the lumbar spine showed no compression of the spinal cord/cauda equina or exiting nerve roots and no abnormal enhancement within the vertebral bodies. MRI of the brain and time of flight angiography (TOF MRA) were unremarkable. A repeat cervical spine radiograph showed no instability, and the implant was in situ.

The patient was treated conservatively with pregabalin. He was referred to Rehabilitation Medicine for further therapy and medication titration.

## Discussion

Paralysis after spinal surgery has multiple causes, including compression of the spinal cord resulting from poorly implanted fixation, hematoma formation, spinal cord edema, and ischemic reperfusion injury, also known as white cord syndrome (WCS). WCS is a very rare surgical complication that is a diagnosis of exclusion, characterized by an ischemic lesion of the spinal cord after spinal decompressive surgery. Although the mechanism of the disease is unclear, it is postulated that ischemic reperfusion injury occurs when blood flow is restored to previously ischemic tissues and organs, causing the formation of oxygen-free radicals that damage the spinal cord and a disruption of the blood-spinal cord barrier [1,2]. There is also an increase in the levels of inflammatory cytokines tumor necrosis factor-α (TNF-α) and interleukin-1β (IL-1β) post-decompression, which was demonstrated in a rat model that had chronic severe spinal cord compression [3]. The occurrence and severity of WCS correlate closely with tissue ischemia time, the extent of ischemic tissue, and the affected tissue’s oxygen requirements [4].

The diagnosis of WCS is characterized by hyperintensity on T2-weighted MRI. Although there have been very few cases of WCS around the world, diagnostic criteria have been formulated, which comprise severe spinal cord compression, surgical decompression, paralysis occurring within three hours post-surgery, motor and sensory dysfunction from the lower to the upper limb, exclusion of other causative factors, and completely or partially restored neurological function by timely high-dose methylprednisolone combined with dehydration and neurotrophic drugs [5]. Our patient had severe stenosis of bilateral C6/C7 neural foramen and developed sensory and motor deficits on postoperative day 6 following C6/C7 ACDF. Many of the earlier reports show an early or near-immediate change in postoperative neurology. In this case, it was postulated that the delayed presentation at postoperative day 6 was due to ischemic reperfusion as the patient had chronic cord compression prior to surgery. A similar case was reported whereby a 59-year-old male presented with neck pain with shoulder radiation, and lower back pain with pain radiating to both legs and was diagnosed as Nurick grade 3 with cervical radiculopathy. The patient underwent a C4-C5 and C5-C6 anterior cervical decompression and fusion (ACDF). Post-ACDF, the patient was only able to move his arms and demonstrated C6 incomplete tetraplegia. At the 16-month follow-up, the patient demonstrated a Nurick grade 4, making his condition worse off than it once was before the ACDF [2]. While these criteria exist, the duration of three hours post-surgery can be debatable. In this case, this patient had a late-onset WCS because of his endothelial damage and atherosclerosis caused by chronic hypertension. A decrease in nitric oxide (NO) precursors led to subacute reperfusion to the compressed spinal area, which led to his late-onset WCS, which occurred 24 hours postoperatively [6].

Many different treatment strategies have been proposed for WCS. The most important is the administration of high-dose methylprednisolone within eight hours of injury, according to the second national acute spinal cord injury study [7]. Steroids upregulate anti-inflammatory factors and decrease oxidative stress, enhancing endogenous cell survival in animal models of spinal cord injury. It reduces edema, prevents intracellular potassium depletion, and inhibits lipid peroxidation. However, an important point to note is that just methylprednisolone may not be enough. Our patient was treated conservatively with pregabalin and physical therapy. In another similar paper, a 71-year-old female had ossification of the posterior longitudinal ligament and underwent a laminectomy, following which she had WCS. Methylprednisolone was not adequate, and restoration of function only returned when posterior instrumented fusion was done [8]. Hence, along with the infusion of steroids, additional decompression may be needed once WCS is diagnosed. The American Association of Neurological Surgeons and Congress of Neurological Surgeons joint guidelines also recommend maintaining a mean arterial pressure (MAP) of at least 85 mmHg for patients with spinal cord injuries [9]. Since the pathophysiology of WCS is proposed to be damage caused by oxygen free radicals, the use of potent antioxidants has also been recommended in the management of WCS [10]. CSF pressures may also have a role to play in the management of WCS.

Preoperative and intraoperative measures can be taken to reduce the risks of WCS. Firstly, remote ischemic preconditioning (RIPC) has been proven to significantly reduce the risk of WCS. A prospective randomized controlled study of adult patients undergoing decompression surgery sought to investigate whether RIPC would protect the spinal cord from ischemic injury. This consisted of three five-minute cycles of upper right limb ischemia with intervening five-minute periods of reperfusion. Neuron-specific enolase and S-100B levels were measured in serum at set time points. The study proved that RIPC significantly reduced S-100B and neuron-specific enolase release [11]. Intraoperatively, electrophysiological monitoring of somatosensory evoked potentials (SSEPs) and motor evoked potentials (MEPs) can be used for spinal surgery [12]. A loss of intraoperative signals is suggestive of direct cord injury, compression, or ischemia. The administration of propofol has also been found to be useful. Propofol decreases histological damage to the spinal cord and reduces the permeability of the blood-spinal cord barrier by downregulating the protein expression levels of nuclear factor-κB (NF-κB) [13].

The timing of decompression also plays a role in the development of WCS. Delayed decompression can exacerbate reperfusion injury and is associated with ongoing enhanced levels of cytokine expression, microglial activation, and astrogliosis, while early decompression inhibits the expression of TNF-α [4]. Hence, the earlier the decompression, the less the risks of WCS occurrence.

## Conclusions

White cord syndrome is a very rare but devastating condition that leaves most patients worse off than they once were. Very few cases have been reported around the world, but it should not be taken lightly. Neurological deficits following spinal decompressive surgery are commonly due to hematoma or iatrogenic injury. Unexplained neurological deterioration in the absence of intraoperative injury should raise the suspicion of a white cord syndrome, more so if there is chronic cord compression prior to surgery. MRI is a key tool in diagnosing WCS, and preventive and postoperative measures should be taken to manage WCS. Understanding the pathophysiology has great potential for future aims of the management of WCS. Although there is no standard treatment protocol for WCS, the current mainstay of treatment is high-dose steroids, intraoperative neurophysiological monitoring, and early recognition of postoperative WCS.
